# Tiny seeds, big decisions: Jasmonate-mediated regulation of seed size in Arabidopsis via the SOD7-KLU module

**DOI:** 10.1093/plcell/koaf160

**Published:** 2025-06-14

**Authors:** Nitin Uttam Kamble

**Affiliations:** Assistant Features Editor, The Plant Cell, American Society of Plant Biologists; Indian Institute of Science Education and Research, Thiruvananthapuram 695551, India

More than a biological unit, a seed is a philosophical symbol of life's continuity. It encapsulates the paradox of being miniature, yet immense in potential. The regulation of seed size, shaped by millennia of natural selection and, more recently, human intervention, reflects a delicate evolutionary balance between survival and utility. Larger seeds often ensure better germination and early growth, conferring resilience in harsh environments, while smaller seeds can disperse more widely, increasing a species’ ecological reach. Manipulation of seed size through breeding and biotechnology has profound implications: enhancing yield for global food security, optimizing nutritional content, and expanding industrial applications.

Extensive research indicates that the regulation of seed size is highly complex, requiring the coordinated action of numerous genes, proteins, and phytohormones such as auxin, cytokinins, and abscisic acid. Recent studies have also reported the involvement of jasmonate signaling in seed size regulation (Hu et al. 2021), in addition to its established roles in plant development and stress tolerance. However, downstream molecular mechanisms by which jasmonate signaling regulates seed size remain largely unexplored.

In new work, **Juping Zhang and colleagues (Zhang et al. 2025)** provide insights into a downstream molecular mechanism of jasmonate-mediated regulation of seed size in *Arabidopsis thaliana*. Using reciprocal crosses, they demonstrate that COI1 and JAZ proteins act maternally with opposing effects on seed size: the COI1 receptor exerts a negative effect, while JAZ repressors exert a positive one (Figure). Beyond seed size, these factors were also shown to influence embryo, seedling, leaf, and petal size. A previous study (Liu et al. 2020) also showed that MYC transcription factors negatively regulate seed size, and their transcriptional activities are regulated by JAZ proteins in the jasmonate signaling pathway.

**Figure.**
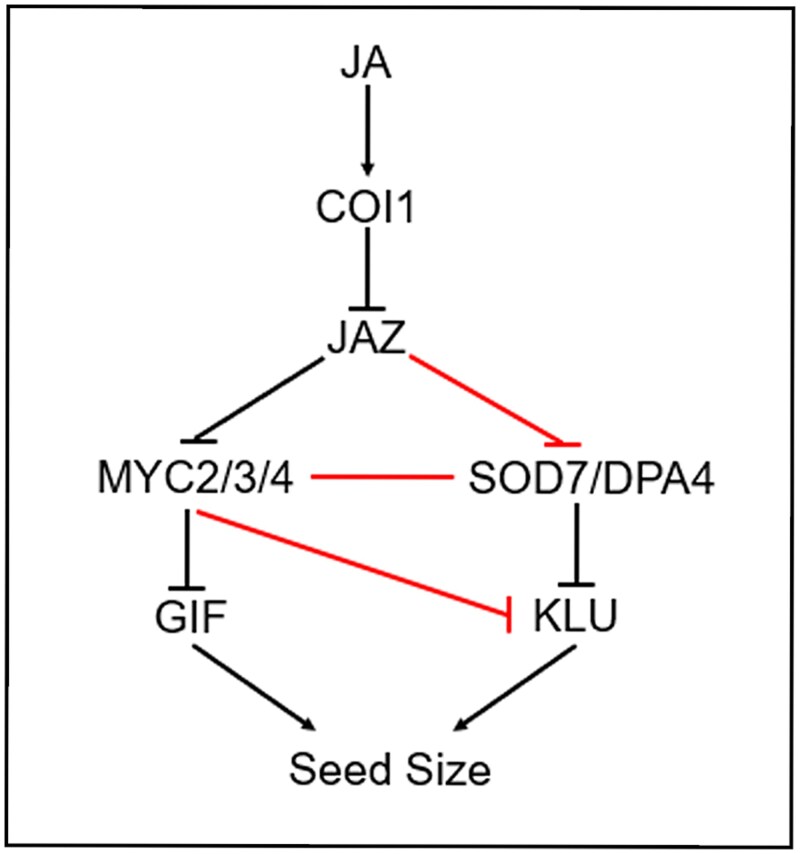
Regulation of seed size by jasmonate signaling in *Arabidopsis* involves COI1, JAZ, SOD7/DPA4, MYC, and KLU. In the presence of jasmonate (JA), COI1 mediates the degradation of JAZ repressors, leading to the suppression of *KLU* expression and inhibition of seed growth via SOD7/DPA4. Additionally, MYC and SOD7 proteins bind reciprocally to regulate *KLU* expression directly. Reprinted from Zhang et al. (2025), Figure 12.

Gene expression and promoter fusion analyses revealed that *COI1*, *JAZ*, and *MYC* exhibit differential expression across developmental stages, particularly in seed integuments. Previous studies established that the cytochrome P450 monooxygenase KLU promotes cell proliferation in seed integuments, with higher expression correlating with larger seeds, while *klu* mutants produce smaller seeds (Adamski et al. 2009). Zhang et al. speculate that jasmonate signaling modulates *KLU* expression. Indeed, *KLU* expression was found to be higher in *coi1/myc234* mutants and lower in *jazQ* mutants. Through genetic crosses between *coi1/myc234* and *klu* mutants, the authors confirmed that *KLU* is required for COI1/mediated repression of seed size.

SOD7 and DPA4 are known repressors of *KLU* and exhibit repressive effects in seed size regulation (Zhang et al. 2015). In this new work, Zhang et al. investigated physical interactions between SOD7/DPA4 and JAZ proteins using various protein-protein interaction assays. They found that the C-terminal regions of SOD7 and DPA4 interact with most JAZ proteins via the ZIM domain. Overexpression of *SOD7* in both wild-type (Col-0) and *coi1* backgrounds resulted in smaller seeds, suggesting that SOD7 is involved in mediating the jasmonate signaling-dependent regulation of seed size and that SOD7/KLU functions downstream of the COI1/JAZ module.

Further mechanistic insights were gained through chromatin immunoprecipitation and dual-luciferase reporter assays, which demonstrated that JAZ proteins interact with SOD7 and interfere with its binding to the *KLU* promoter (Figure). Zhang et al. further examined whether SOD7 also interacts with MYC transcription factors. Protein-protein interaction assays revealed that MYC2 and MYC4 physically interact with SOD7 to mediate enhanced binding of both SOD7 and MYC2 to the *KLU* promoter. Genetic analysis of crosses between *myc234* mutants and *SOD7* overexpressors suggested that MYC and SOD7 act in concert to negatively regulate seed size. JAZ1 was also shown to inhibit the transcriptional functions of both SOD7 and MYC2 to modulate *KLU* expression. Additionally, jasmonate was found to induce the degradation of KLU via the 26S proteasome pathway.

To further elucidate the jasmonate-dependent regulation of seed size, the authors also investigated the effects of salt stress on various mutant combinations. They found that the COI1-mediated jasmonate signaling plays a role in limiting seed size under salinity stress.

In summary, this study elucidates previously unknown molecular mechanisms of seed size regulation via the jasmonate signaling pathway in *Arabidopsis*, highlighting a SOD7-KLU regulatory module. Further research is required to explore the roles of these transcriptional regulators under specific natural environmental conditions and their crosstalk with other signaling pathways (e.g. hormonal pathways) in seed size determination. Understanding these regulatory networks holds significant potential for developing seeds with enhanced agronomic traits.

## Recent related articles in *The Plant Cell*

- Chen et al. (2024) described the role of the OsMOB1A-OsSTK38 kinase complex in the phosphorylation of CYCLIN C, regulating grain size and weight in rice.
- Mei et al. (2023) reported that auxin contributes to jasmonate-mediated regulation of abscisic acid signaling during seed germination in Arabidopsis.
- Varshney et al. (2023) demonstrated the regulation of JAZ proteins during seed maturation and seed vigor, independently of jasmonic acid-isoleucine, by the F-box protein SKP1-INTERACTING PARTNER 31 in Arabidopsis.
- Yuan et al. (2024) identified the genetic basis of seed weight and oil content in soybean through integrative omics analysis.
- Zhou et al. (2023) reported a CYP78As-SMG4-COPII complex module that promotes grain size in rice.

## Dive Curated Terms

The following phenotypic, genotypic, and functional terms are of significance to the work described in this paper:

JAZ1 Gramene: At1g19180
JAZ1 Araport: At1g19180

MYC4 Gramene: AT4G17880
MYC4 Araport: AT4G17880

COI1 Gramene: AT2G39940
COI1 Araport: AT2G39940

MYC2 Gramene: AT1G32640
MYC2 Araport: AT1G32640

DPA4 Gramene: AT5G06250
DPA4 Araport: AT5G06250
